# Mechanical Degradation and Failure Analysis of Different Glass/Basalt Hybrid Composite Configuration in Simulated Marine Condition

**DOI:** 10.3390/polym14173480

**Published:** 2022-08-25

**Authors:** Alex Osei Bonsu, Comfort Mensah, Wenyan Liang, Bin Yang, Yunsheng Ma

**Affiliations:** 1 College of Aerospace and Civil Engineering, Harbin Engineering University, Harbin 150001, China; 2 School of Aerospace Engineering and Applied Mechanics, Tongji University, Shanghai 200070, China; 3 Shandong Chambroad Holding Group Co., Ltd., Binzhou 256500, China

**Keywords:** seawater ageing, hybrid composite, basalt fiber, glass fiber, mechanical properties

## Abstract

This work aims to evaluate the failure mechanisms of plain glass and basalt fiber reinforced composites and a selected glass/basalt hybrid composite sequence subjected to artificial seawater conditions. Sets of plain and five hybrid composite configurations were fabricated by vacuum assisted resin injection technique (VARI), and subjected to seawater aged for 258 days at 30 °C and 70 °C followed by tensile, flexural and charpy impact testing, respectively. Failure analysis for dry and seawater-aged composites were undertaken using scanning electron microscopy (SEM). Results showed that some hybrid laminates with sandwich-like and alternating sequencing exhibited superior mechanical properties and ageing resistance than plain laminates. GB3 ([B2G2]_S_) type hybrid composite with basalt fiber outer plies retained 100% tensile strength and 86.6% flexural strength after ageing, which was the highest among all the laminates. However, GB4 ([BGBG]_S_) type specimen with alternating sequencing retained the highest residual impact strength after ageing. SEM analysis on the failed specimens showed fiber breaking, matrix cracking and debonding caused by fiber–matrix interface degradation due to seawater exposure. However different hybrid configurations to a considerable extent prevented crack propagation across specimens, hence altering the overall damage morphology among different specimens.

## 1. Introduction

There has been an increase in studies on the use of hybrid composites in place of plain composite structures over the years. This phenomenon is due to the hybrid composite possessing properties which cannot be achieved using a single reinforced composite. Usually, the aim of designing hybrid structures is to build a much more robust structure by utilizing the advantages of the constituent reinforcing fibers while relieving each of its individual flaws [1,2,3]. Hybrid composites present several unique benefits over composites made using single fiber reinforcement. First of all, they offer the engineers the ability to produce structures with unique characteristics. Such characteristics may involve enhanced ductility [4], improved mechanical strength/stiffness [5,6], and increased resistance to corrosive substances [7]. Depending on the stacking sequence and fiber orientation, hybrid composites may also offer good impact resistance, and a longer fatigue life [8]. Additional advantages and other drawbacks to using hybrid composites can be seen in Table 1.

In this context, Javaid et al. [9] investigated the static and fatigue properties of hybrid glass/carbon fiber composite joints with different layups. His findings showed different hybrid sequences performed well under different conditions. For example, specimens with glass on the outside resulted in better performance under static tension and fatigue. However, the interleaving hybrid specimens exhibited good tensile performance but were the worst regarding fatigue. Jagannatha and Harish [10] also proved that introducing carbon fibers into glass fiber layers resulted in enhanced micro-hardness and improved tensile properties of the hybrid specimens.

Some articles have also touched on various environmental effects on hybrid composites. Among these studies, the thermomechanical properties of hybrid composites were evaluated by Tsai et al. [11]. Their results showed a slowdown in the property degradation of the hybrid composite after the introduction of carbon fiber in the hybrid composite. This was attributed to the lower moisture absorption property of the hybrid system, which is also similar to the findings of Dhakal et al. [4]. Studies by Tomasz et al. [12] on the residual mechanical strength of glass/carbon hybrid composite bars in harsh environments showed that hybrid composites resisted aggressive environments better than plain composites. They attributed the results to the filament-wound glass layer, which served as a form of protection to the carbon fiber core. However, specimens with glass fiber cores and carbon fiber overwraps showed delamination of the interface and degradation of the core structure from the outer layer. Amini et al. [13] also assessed the tensile failure mechanisms of environmentally degraded hybrid glass/carbon fiber reinforced epoxy composites and found matrix cracking, fiber–matrix interface debonding, and fiber failure to be the leading causes of strength degradation.

The current trajectory and environmental concerns mean more emphasis has been placed on the use of and research into natural or more environmentally friendly fibers [6]. The non-synthetic basalt fiber is acquired from volcanic rock and possesses comparable characteristics to S-2 Glass fiber. It offers exceptional properties, such as good fire resistance, high tensile strength, good fiber–resin adhesion, chemical stability and good corrosion resistance. Several researchers have reported on the use of basalt fiber as reinforcement. Among these researchers, Liu et al. [14] explored the use of basalt fibers in the field of transportation and concluded that both glass and basalt fiber specimens possessed similar properties. Subsequent work from the same authors focused on the environmental durability of glass and basalt fiber-reinforced polymer composites. Possible environmental forces, such as sea water exposure, moisture absorption, temperature and moisture cycling, were thoroughly investigated [15]. Results after 240 days ageing showed a decrease in both tensile strength and Young’s modulus of the test specimens. Similar works by Wei et al. [16] showed that glass and basalt epoxy resin, after long-term seawater ageing, may experience some irreversible physical or chemical degradation.

From the aforementioned literature, it can be seen that the existing literature on hybrid composites focuses on combining high-strength/low-strain materials, such as carbon fiber, with lower-strength fibers to substitute their individual drawbacks [17,18,19]. However, solutions to issues of damages induced due to stress concentration emanating from the low strain material have been largely ignored. In addition, scholars emphasize mechanical properties in relation to the stacking sequence of fibers, but few studies have focused on interface degradation of the different hybrid composites resulting from seawater exposure [20]. 

Basalt fiber for the most part has comparable properties to glass fiber but specific characteristics such as high chemical stability [21], little to no toxins and resistance to high temperatures [22], makes it an ideal material for hybridizing with E-glass in marine applications. Although previous studies focused on hybridizing extremely dissimilar materials, we believe using materials with similar mechanical properties, like glass and basalt fiber, could also provide good outcomes by reducing issues of stress concentration encountered when using carbon fiber. The added chemical stability of basalt fiber could also offer additional benefits for the hybrid materials in a chloride environment. 

In this article the ageing resistance of plain glass and basalt reinforced polymer composite is compared with glass/basalt hybrid composite prepared with different stacking sequences. In total, five types of hybrid composite configurations (i.e., [G_3_B]_S_, [G_2_B_2_]_S_, [B_2_G_2_]_S_, [BGBG]_S_, [GB_3_]_S_) were fabricated by the vacuum assisted resin injection technique and seawater aged for 258 days at 30 °C for tensile/flexural specimen and 70 °C for impact specimens. Mechanical properties of the composites were investigated pre-ageing and after ageing using tensile, flexural and charpy impact tests in accordance with ASTM standards. Furthermore, failure analysis for dry and seawater aged composites were undertaken using scanning electron microscopy (SEM).

**Table 1:** Advantages and disadvantages of hybrid composites.

Advantages	Disadvantages	Ref.
They offer a cost-cutting solution to manufacturers.	The final hybrid composite might be slightly less durable depending on the hybrid materials used.	[23]
Improved high thermal and electrical conductivity than the constituent material.	Reduced thermal and electrical conductivity than the constituent material.	[24]
Natural fiber reinforced hybrid composites are environmentally friendly and sustainable.	Addition of natural fibers can reduce the overall strength of the hybrid composite.	[25]
Reduced moisture absorption capability than the constituent fibers,	Can increase the moisture absorption properties of the original materials.	[26]

## 2. Materials and Methods

### 2.1. Materials Properties

Unidirectional E-glass and unidirectional basalt, both with surface density of 300 g/m^2^, were used separately as the composite’s reinforcement. These fibers were selected for their similarities in mechanical properties. This prevents issues of stress concentration after fracturing in, for example, low-strain carbon fiber wrapped around high strain-glass fibers. Both fibers used in this study were procured from Yixing Huaheng high performance fiber textile Co. Ltd., Yixing, China. The fibers were used in conjunction with Bisphenol A, DERAKANE411–350 epoxy vinyl ester resin which has 45 wt% styrene (phenylethylene) and 350 cps viscosity and can be cured under ambient temperature. Methyl Ethyl Ketone Peroxide (MEKP) was used as the hardening agent and paired with Dimethylaniline as the accelerating agent at a mix ratio of 1:1%:0.1% by weight. Both materials and the matrix were procured from Harbin Akihito composite matrial Co.Ltd., Harbin, China. This resin was selected based on characteristics such as superior corrosion resistance, chemical stability and, also, better moisture absorption resistance than polyester [27]. The vendor supplied specifications of the materials can be seen in Table 2.

### 2.2. Composite Fabrication

**Figure 1:**
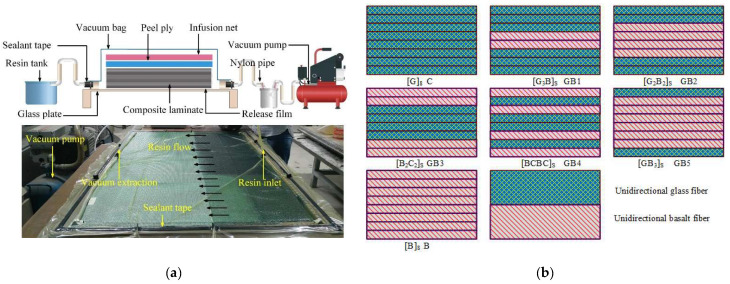
(**a**) Photograph and schematic illustration of VARI processing and (**b**) stacking sequence of laminates.

Details of the Vacuum assisted resin injection (VARI) [28,29] in inlet process can be seen in Figure 1. We began the specimen manufacturing process by first assembling eight layers of fibers with the aim of achieving our desired laminate thickness. This was followed by coating the glass base plate with a releasing agent. The selected plies were carefully assembled on the mold, followed by peel ply, infusion net and, finally, a vacuum bag. The entire mold was then sealed using a sealant tape and debulked. Hardening and accelerating agents were evenly mixed with the matrix at a given ratio. This step was followed by placing the suction tube into the resin mixture, which gradually soaked up the entire prepared fabrics. The injection tube was immediately sealed after the resin injection, allowing the preforms to cure overnight at room temperature. Seven composite configurations were manufactured using the above-mentioned process. As seen in Figure 1b, the structural configurations were [G]_8_, [G_3_B]_S_, [G_2_B_2_]_S_, [B_2_G_2_]_S_, [BGBG]_S_, [GB_3_]_S_, [B]_8_ which were also represented as G, GB1, GB2, GB3, GB4, GB5, B.

### 2.3. Ageing Process

**Figure 2:**
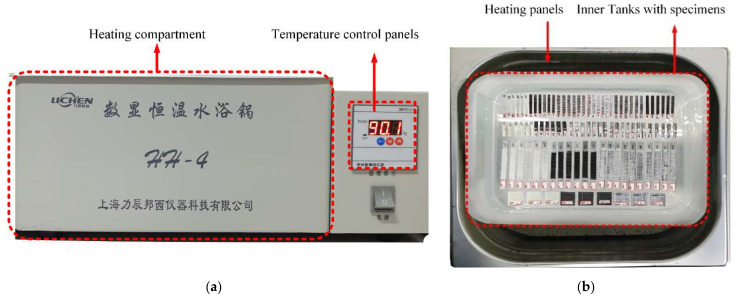
Photograph of ageing chamber (**a**) outside (**b**) Inside.

Before ageing, the prepared laminates were dried repeatedly in an oven every 24 h at 50 °C. This was done to achieve a stable mass for both specimens before proceeding to the next phase. Five specimens of each material were prepared for ageing in the climate chambers, as shown in Figure 2. The default temperature for the ageing chamber ranged from a minimum of 30 °C to a maximum of 100 °C and was able to hold up to 20 L maximum capacity of water. The composite laminate samples were immersed in artificial seawater (with salinity of about 3.5%) solution in the sealed chamber at 30 °C and 70 °C for 258 days. This ageing duration was chosen to enable the aged composites to undergo chemical degradation (hydrolysis), which took place after 90 days of ageing.

### 2.4. Mechanical Tests

#### 2.4.1. Tensile Test

**Figure 3:**
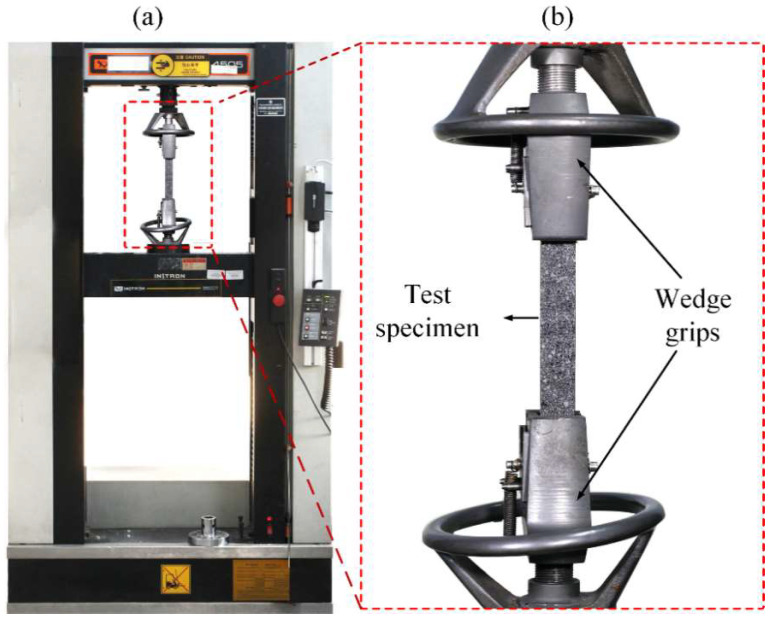
Photograph of tensile testing configuration (**a**) Instron testing machine (**b**) Tensile test specimen.

Laminates for the tensile tests were cut into 250 mm × 25 mm × 1.9 ± 0.2 mm. This dimension was chosen in accordance with the ASTM D3039M-17 standard. The Instron Universal Testing was used for tensile testing with a loading rate set to 2 mm/min, as shown in Figure 3. Five test specimens for each group were used during testing. From the test data, stress/strain, tensile strength, Young’s modulus, strength retention and failure strain of control and aged composites were determined. Tensile properties are given by:

(1) $$\sigma =\frac{F}{A}$$

(2) $$\varepsilon =\frac{\Delta L}{L}$$

(3) $$E=\frac{\sigma }{\varepsilon }$$

where σ, ε and E represent the stress, strain and modulus. F is the force in newton. *A* is the cross-sectional area ΔL is the displacement in mm and L is the gauge length. 

#### 2.4.2. Flexural Test

**Figure 4:**
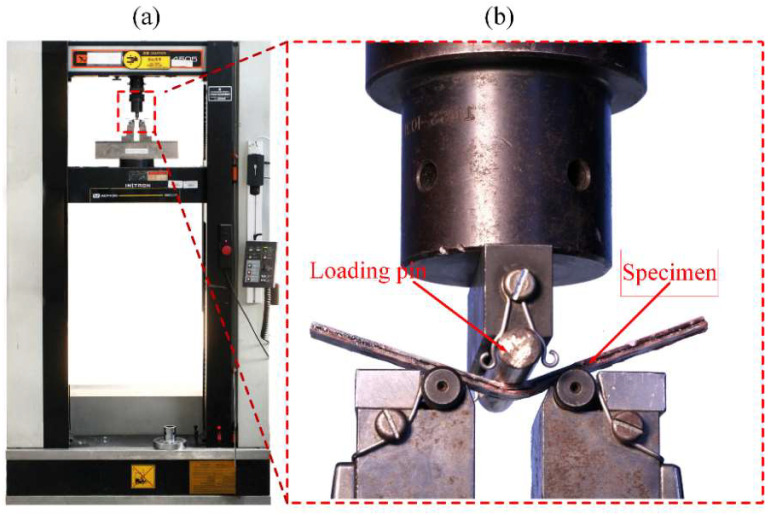
Photograph of flexural testing configuration (**a**) Instron testing machine (**b**) Flexural test specimen.

Figure 4 shows the setup for flexural testing. A three-point bending test with loading rate of m/min was conducted in accordance with ASTM D7264M-15. Each sample category comprised five rectangular specimens cut from composite laminate with dimensions of 120 mm× 15 mm× 2 mm. Using the Zwick/Roell testing machine, specimens were placed in the center of the fixture with a 6 mm diameter loading nose and span-to-thickness ratio (S:T) of 32:1. Flexural properties were given by:

(4) $$\sigma_{F}=\frac{3P_{max}L}{2wt^{2}}$$

(5) $$E_{F}=\frac{mL^{3}}{4wt^{3}}$$

(6) $$\varepsilon_{F}=\frac{6dt}{L^{2}}$$

where$\;\sigma_{F}$*,* $\varepsilon_{F}$ and $E_{F}\;$represent the flexural strength, modulus and flexural strain. L is the span length, w is the width and t is the thickness of the test samples. $P_{max}\;$is the maximum load before failure, m is the slope of the initial segment of load versus displacement curve, d is the maximum bending before failure.

#### 2.4.3. Impact Test

**Figure 5:**
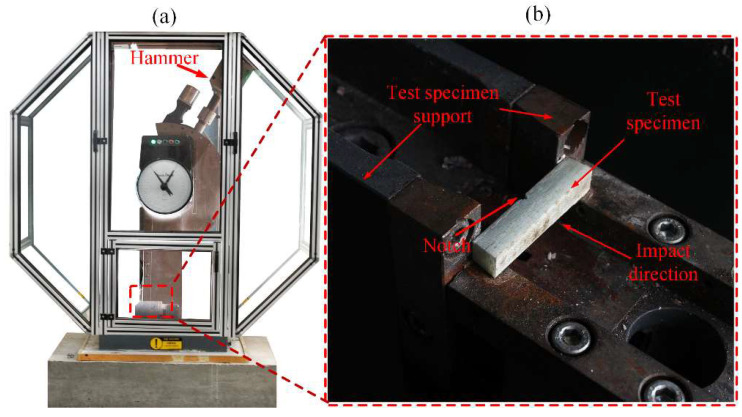
Photograph of Charpy impact testing configuration (**a**) Testing machine (**b**) Charpy impact test specimen.

Charpy impact testing was undertaken with a Zwick/Roell Charpy impact testing machine, as seen in Figure 5. This devise is equipped with a 300 J Hammer. The specimens were a group of v-notched laminates with 55 mm (length) × 10 mm (width) × 10 mm (thickness) as the dimensions following the recommendation of the ASTM D6110–17 standard. During testing, the samples were carefully placed in cantilever position, while the impactor swung across a pendulum direction to break the specimen. Both impact strength and strength retention were determined after the test, while closely monitoring the different fracture morphology for both materials after impact.

### 2.5. Damage Analysis

Failure mechanism of both plain and hybrid composites was analyzed using Scanning electron microscope (SEM). Additionally, analysis on the interaction between water molecules and the matrix was carried out using Fourier-transform infrared (FTIR) spectroscopy. Aged and unaged specimens of 1 × 1 cm were used for this test. During the test, samples were pressed into a pellet with potassium bromide (KBr) and scanned from 4000 to 500 cm^−1^ at a resolution of 4 cm^−1^.

## 3. Results and Discussion

### 3.1. Tensile Properties of Dry and Seawater Aged Laminates

**Figure 6:**
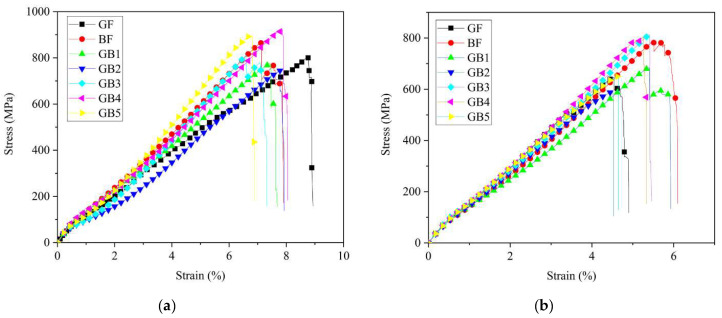
Tensile stress-strain curves of (**a**) dry specimens (**b**) seawater aged specimens.

A summary of the tensile properties of dry and aged composites can be seen in Table 3 and Table 4. The subsequent stress versus strain curves are shown in Figure 6a,b. It is clear from Figure 6a that the stress/strain curve for plain composites exhibited a linear elastic region followed by nonlinear section up to a maximum point, then sudden catastrophic failure occurred. Plain B composite showed higher tensile strength and lower failure strain in dry conditions compared to plain G composite. Tensile strengths of GB1, GB2 and GB5 hybrid composites increased with an increase in basalt fiber core layers; however, the failure strain decreased under the same conditions. A similar report by Pandya et al. [30] also showed improvement in tensile strength of hybrid composites due to the addition of higher strength fiber layers in the core.

The tensile strengths of GB2, GB3 and GB4 revealed that hybrid composites with plain glass fiber skin had lower strength but higher strain at break than GB3 with basalt skin. GB4, on the other hand, had the highest tensile strength and slightly higher failure strain relative to the other composites. These tensile strength and failure strain characteristics displayed by hybrid composites are due to the bridging effect of the high elongation fiber and its support to the broken brittle fiber in the hybrid laminates [31]. Kretsis [32] also reached a similar conclusion, revealing that the weakest fiber in a hybrid composite is usually the first to fracture, causing stress concentration around the fractured fibers. The surrounding stress then hastens crack propagation. However, the adjoining high strain fiber layers would bridge the fractured low strain fiber to prevent propagation of the cracks. This phenomenon then allows the stronger fibers to reach their ultimate strength before final failure. For most of the hybrid specimens there was an almost linear decline after reaching the peak load, as seen in Figure 6a. In a nutshell, there was not a significant load drop and recovery with the fracture of the low and high elongation fibers. This was a result of the similarities in the properties of these materials. This is in contrast with the result of Czél et al. [33] who experienced a significant load drop due to the use of hybrid materials with significantly different properties.

**Figure 7:**
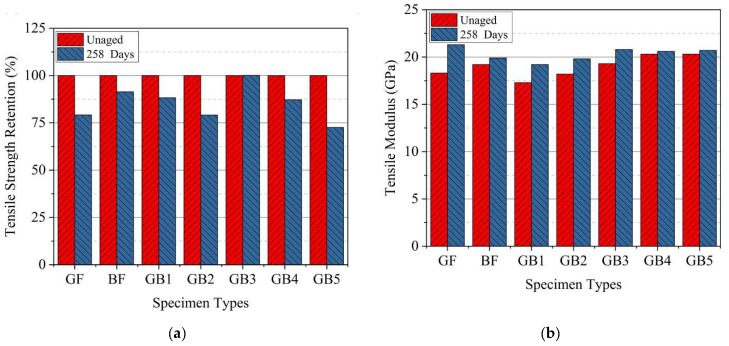
Tensile (**a**) strength retention (**b**) modulus of specimens in dry and seawater aged condition.

As seen in Figure 6b and Table 4 the tensile strength and failure strain results after seawater ageing decreased for all the composites, except for a slight increase in strength for GB3 specimen. Tensile strengths of aged G, B, GB1, GB2, GB3, GB4 and GB5 reduced by 21%, 9%, 12%, 21%, 0%, 13%, and 27%, respectively. B composite retained a higher tensile strength after ageing than G composite while its failure strain surpassed that of the latter, as seen in Figure 7a. This might be due to higher moisture absorption properties of basalt fiber. Test results from GB1, GB2, and GB5 hybrids showed higher tensile strength retention with an increase in outer glass fiber layers. A comparison of the results from GB2, GB3 and GB4 composites also showed the highest strength retention for specimens with an outer basalt fiber layer. The reason for these results could be the lower water absorption properties of the outer glass fiber layers and, also, the chemical stability of the basalt fiber layers after seawater treatment. These properties help maintain a better fiber–matrix interfacial adhesion. Figure 7b shows the variation of the flexural modulus for plain and hybrid composites after seawater ageing. It is worth noting that, for the given ageing duration, both plain laminates and the hybrid configurations all showed a slight decrease in tensile modulus after seawater ageing, except GB3 composite.

#### Tensile Failure Analysis

**Figure 8:**
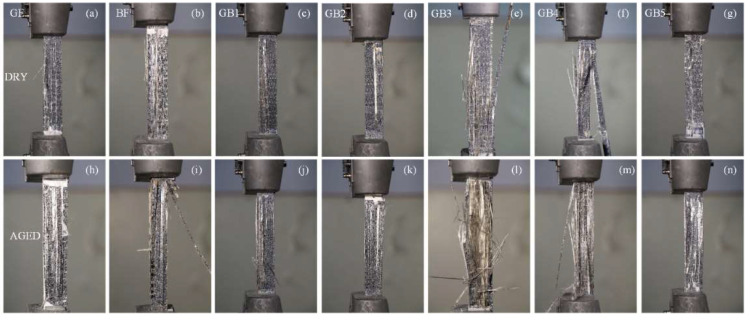
Photograph of tensile test specimens in dry (**a**) GF (**b**) BF (**c**) GB1 (**d**) GB2 (**e**) GB3 (**f**) GB4 (**g**) GB5 and seawater aged condition (**h**) GF (**i**) BF (**j**) GB1 (**k**) GB2 (**l**) GB3 (**m**) GB4 (**n**) GB5.

Figure 8 shows the failure mode of dry and seawater aged specimens. Longitudinal splitting along the loading direction was observed, while fracturing started around the gauge length and slowly advanced towards the direction of the applied load. From Figure 8a, G composite (plain glass fiber composite) exhibited ductile fracture under tensile loading. In contrast, B composite (plain basalt composite) showed a slightly more brittle fracture in tension than G composite as seen in Figure 8b. Meanwhile from the hybrid composite, the failure morphology consisted of fracturing of fibers, matrix cracking and delamination. The delamination within the glass/basalt interface was due to the weak adhesion between the different plies as seen in Figure 8c–g. Similar damage mode to the aforementioned ones with severe debonding between layers and pull outs were observed after ageing, indicating the severity of degradation in mechanical properties as seen in Figure 8h–n.

**Figure 9:**
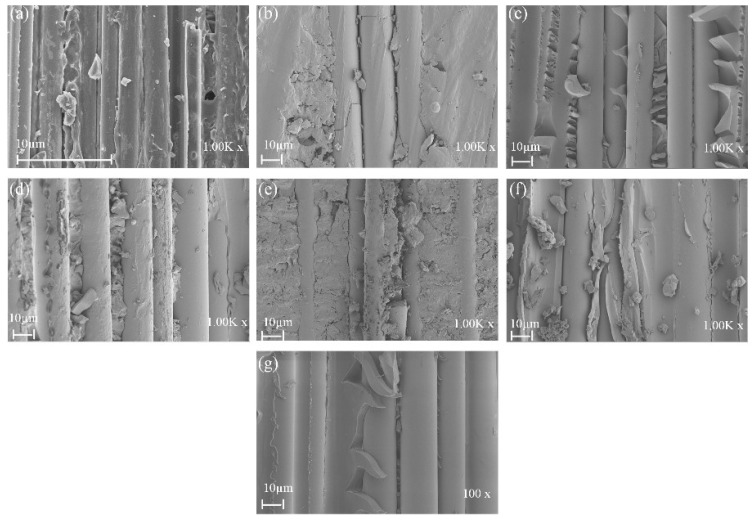
SEM images of tensile specimens in dry condition (**a**) GF (**b**) BF (**c**) GB1 (**d**) GB2 (**e**) GB3 (**f**) GB4 (**g**) GB5.

**Figure 10:**
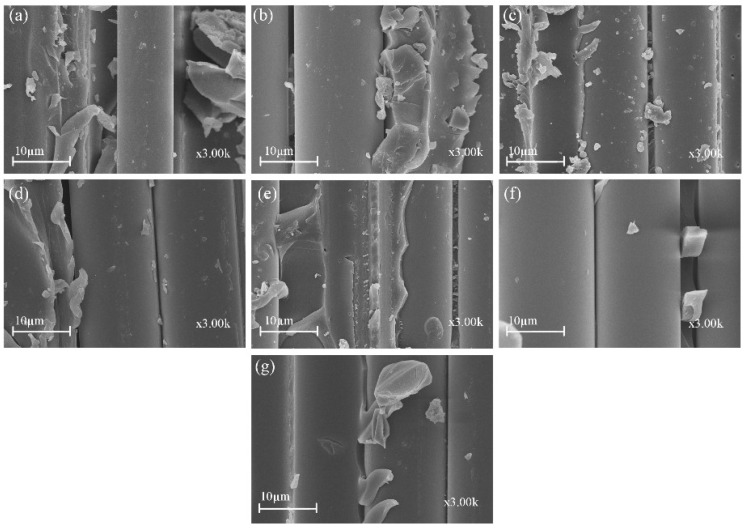
SEM images of tensile specimens in seawater aged condition (**a**) GF (**b**) BF (**c**) GB1 (**d**) GB2 (**e**) GB3 (**f**) GB4 (**g**) GB5.

Figure 9 and Figure 10 show the SEM of tensile damage morphology in dry and seawater aged conditions. From Figure 9a, the typical failures of G composite included fiber fracture and pull-outs. It was also seen that a decent amount of resin still remained wrapped in some parts of the fibers, which was indicative of good fiber–matrix interfacial bond. In Figure 9b, however, B composite is shown to have had a better interface bond with the matrix, resulting in shear cusp formation and more residual resin on the laminate. The SEM images of failed GB1, GB2 and GB5 composites are shown in Figure 9c,d,g. Shear cusp formation and delamination were the predominant damage mode for this group of specimens. This could be attributed to the brittleness of the resin matrix and mismatch between dissimilar core and outer layers of the hybrid composite. For GB3 and GB4 composites, with outer basalt fiber layers, a higher amount of resin wrap was present, as seen in Figure 9e,f. The residual resin showed that basalt fiber in the exterior layers could alter the failure modes of the composites. Figure 10a,b show the typical failure of plain glass and basalt fiber laminates after seawater ageing. A higher degree of debonding and imprints, due to interface degradation, can be seen for both specimens compared to the dry laminates. Similar damage modes were observed for the hybrid composites, as shown in Figure 10c–g.

Results for FTIR spectra of G and B composites in dry and different ageing durations can be seen in earlier studies [34]. Results from this test show chemical change in specimen after ageing. The result showed an obvious change in spectra for both materials, which was an indication of the chemical change after seawater treatment. From the hydroxyl regions (3800–2500 cm^−1^), O-H stretching of the hydroxyl group and stretching vibration of the C–H bond decreased in intensity. These could be attributed to moisture absorption and hydrolysis reaction of the resin matrix. A decrease in the fingerprint peaks was also observed. Amin Khajeh et al. [35] attributed this phenomenon to dehydration following hydrolysis reaction.

### 3.2. Impact Properties of Dry and Seawater Aged Laminates

**Figure 11:**
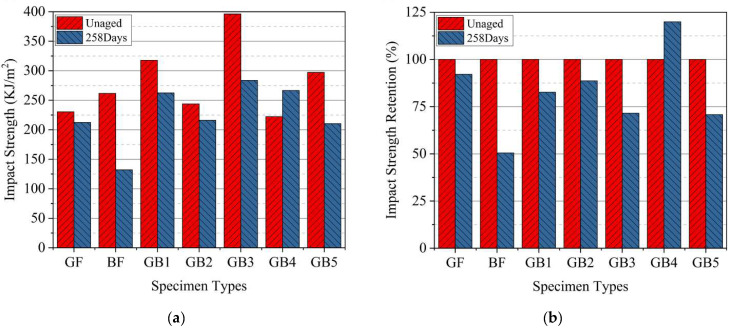
Impact (**a**) strength (**b**) strength retention of specimens in dry and seawater aged condition.

In determining the impact properties or toughness of composite laminates, several factors, like geometry of the specimen, the layering sequence of the laminate, fiber–matrix interface and test conditions play very important roles [36]. In this study, the impact strength of dry and seawater aged composites were evaluated and the results are presented in Figure 11 with subsequent data in Table 3 and Table 4. The results show that the impact strength of plain B composite was higher than G composite in dry state. However, the impact strength of dry GB1 and GB2 increased with an increase in glass fiber outer layers, regardless. GB5, with higher basalt core and lower glass skin, surpassed both composites in strength, as seen in Figure 11a. The impact strength of GB2, GB3 and GB4 revealed that hybrid composites with plain glass fiber skin had lower strength than GB3 with basalt fiber skin. GB4, with alternating hybrid sequencing, had the lowest strength in this group but was higher than both plain composites. This shows that adding some amount of glass or basalt fibers to the core in hybrid composites can better improve the impact resistance of the composites over alternating fiber sequencing in GB4, thus reinforcing the vital role of different hybrid fiber sequencing [36].

The absorption of water resulted in the deterioration of impact strength after ageing, as seen in Figure 11b. From the diagram it can be seen that the G composite maintained better impact strength after seawater ageing than B composite. The impact strength of seawater aged B and G composites decreased by 49.52% and 7.88%, respectively. The results from GB1, GB2, and GB5 hybrid composites, however, showed a higher impact strength retention for GB2 laminate with a medium amount of glass fiber outer layers. A comparison of the results from GB2, GB3 and GB4 composites, on the other hand, showed the highest impact strength retention for specimens with alternating hybrid sequencing.

#### Impact Failure Analysis

**Figure 12:**
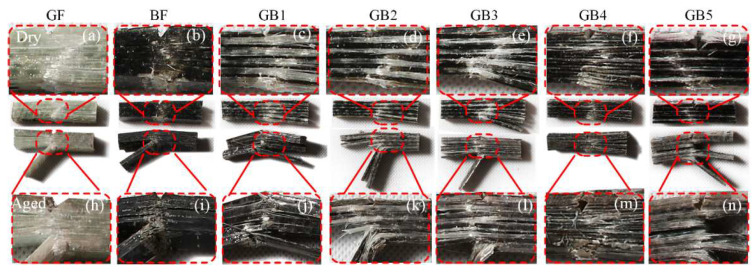
Photographs of impact fractured specimens in dry (**a**) GF (**b**) BF (**c**) GB1 (**d**) GB2 (**e**) GB3 (**f**) GB4 (**g**) GB5 and seawater aged condition (**h**) GF (**i**) BF (**j**) GB1 (**k**) GB2 (**l**) GB3 (**m**) GB4 (**n**) GB5.

Charpy impact testing for composite laminates normally produces failure mechanisms, such as complete break, hinge break, partial break and non-break. The failure mode of the dry specimen was mostly partial to non-break, while that of an aged specimen was mostly a combination of the above failure modes, as seen in Figure 12. The ductile nature of G composite resulted in less break after impact testing as seen in Figure 12a. This damage mechanism stemmed from the energy absorption capability of the high strain glass fiber resulting in more deformation but less break compared to the relatively lower strain B composite seen in Figure 12b. The plain basalt composite, however, broke in the impact zone with less deformation. From Figure 12c–g it can be observed that, the hybrid composite damage was a combination of the effects of G and B failure morphologies. For aged laminates, however, there was swelling and degradation of the matrix, which resulted in less deformation and much more fracturing after impact, as seen in Figure 12h–n.

**Figure 13:**
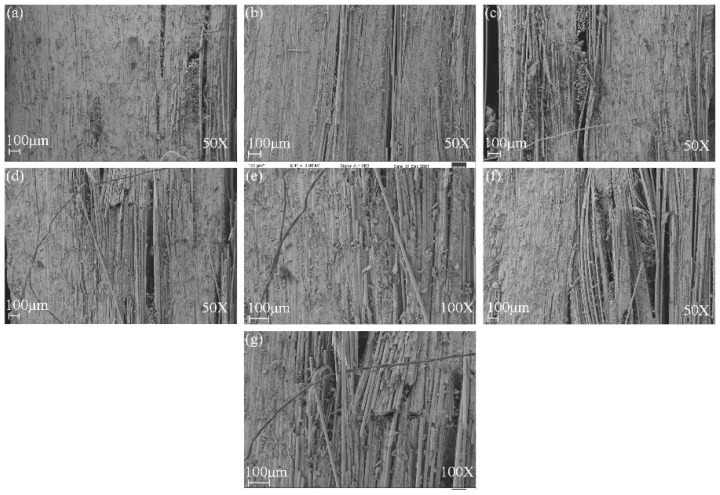
SEM images of impact specimens in dry condition (**a**) GF (**b**) BF (**c**) GB1 (**d**) GB2 (**e**) GB3 (**f**) GB4 (**g**) GB5.

**Figure 14:**
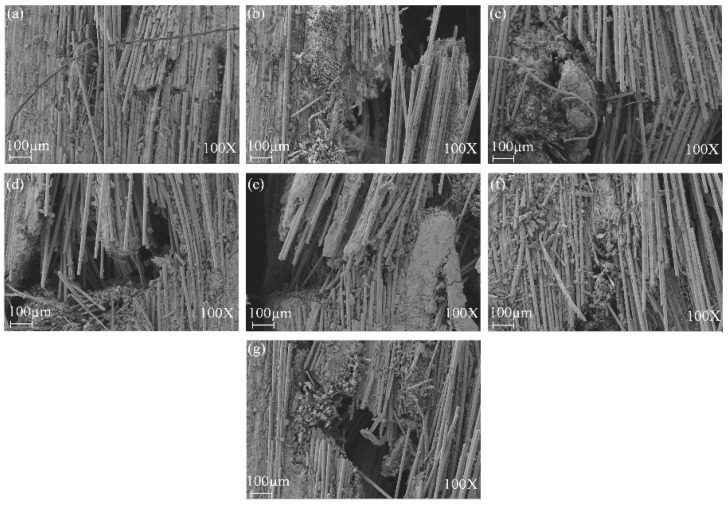
SEM images of impact specimens in seawater aged condition (**a**) GF (**b**) BF (**c**) GB1 (**d**) GB2 (**e**) GB3 (**f**) GB4 (**g**) GB5.

Figure 13 shows the SEM damage morphology of plain/hybrid composites in dry condition. From Figure 13a,b the typical failures of plain glass and basalt laminate included delamination and a few debonding of fibers prior to seawater ageing. It was also seen that a decent amount of resin still remained wrapped in some parts of the fibers, which was indicative of a good fiber–matrix interfacial bond. In Figure 13c,d,g, however, an increasing B core led to increasing damage in the laminate core layers while Figure 13e,f show damages in skin and core layers, respectively. After seawater ageing, however, the damage modes of the composite followed a similar pattern, as seen in Figure 14a–g. Plain glass and basalt fiber laminates after seawater ageing both had hinge breaking with a higher degree of fiber fracture for B compared to G specimen. Higher degrees of debonding and imprints, due to interface degradation, can be seen for both specimens compared to the dry laminates. Damage mode for hybrid structures were also complete to partial breaking, debonding and fracturing of basalt core in the hybrid structure as seen in Figure 14c–g.

### 3.3. Flexural Properties of Dry and Seawater Aged Laminates

**Figure 15:**
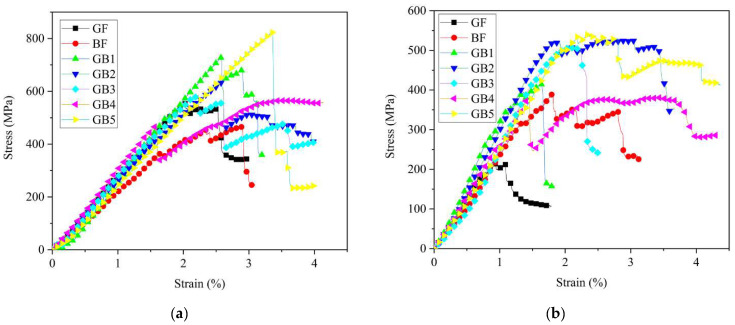
Flexural stress-strain curves of (**a**) dry specimens (**b**) seawater aged specimens.

Figure 15a,b show the flexural stress versus strain curves of dry and seawater aged composites with supporting data in Table 3 and Table 4. It can be seen that the laminates initially presented a linear elastic behavior. At this stage, the fibers and matrix bore the load with no obvious fracture. However, gradual loss in load bearing ability was observed in the subsequent stage, due to formation of cracks in the matrix. Regardless, the reinforcing fibers still carried the load until the onset of compressive failure in the upper ply. For dry conditions, it was observed that plain G composite had a higher flexural strength than plain B composite, as seen in Figure 15a. Besides, the flexural strengths of dry hybrid composites also showed significant differences. Flexural strength and failure strain of GB1 and GB2 increased with an increase in glass fiber outer layers. However, GB5 with higher basalt core and lower glass skin, surpassed both composites in strength and strain. This was because the outer glass layers bore higher load under flexure. Interestingly, non-linear behavior was observed for most of the specimens after reaching the maximum stress. Similar results in [37] were also the result of replacing the outer carbon layers with high elongation basalt layers. 

The flexural strength of GB2, GB3 and GB4 revealed that hybrid composites with plain glass fiber skins had higher strength than GB3 and GB4 with basalt fiber skins. Besides, GB4, with alternating hybrid sequencing, had the lowest strength in this group but was higher than both plain composites. Similar results from Zhang et al. [31] showed improved flexural strength for hybrid specimens with higher strength outer surfaces like carbon and a comparatively higher elongation glass fiber as core. However, in our case glass fiber reinforced composite was higher in strength than basalt. Additionally, the improved flexural strength of the hybrid composite, in comparison with the plain laminates, could be explained by the fact that, after fracturing in the compressive ply, further damage propagation across the laminate was delayed by the inserted core layer. This allowed a gradual loss in load carrying ability on the compressive side, while the tensile side bore the load till final failure. This steady failure could act as an early warning indicator which was contrary to the failure mechanism of plain composites

**Figure 16:**
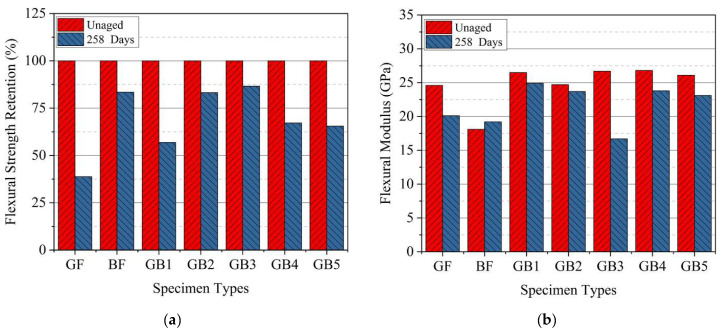
Flexural (**a**) strength retention (**b**) modulus of specimens in dry and seawater aged condition.

Overall, the flexural strength after seawater ageing decreased for all the composites, as seen in Figure 15b. The flexural strength of aged G, B, GB1, GB2, GB3, GB4 and GB5 reduced by 61%, 16%, 43%, 16%, 13%, 32%, and 34%, respectively, compared to dry conditions, as seen in Figure 16a. Strength reduction in G composite after ageing was higher than B composite, probably due to the chemical stability of basalt composite which helped maintain the fiber-matrix interfacial adhesion after ageing. Comparing the results from GB1, GB2, and GB5 hybrid composites it can be seen that higher flexural strength retention was recorded with an increase in glass fiber outer layers. However, GB5 did not follow the same trend, as it recorded the highest strength reduction of the three specimens after ageing. A comparison of the results from GB2, GB3 and GB4 composites also showed the highest strength retention for specimens with an outer basalt fiber layer; thus, GB3 composite. Meanwhile GB4 specimen showed the lowest strength retention among the group after ageing. The reduction in flexural strength was mainly caused by swelling and degradation of matrix after seawater exposure [38]. This caused weakness in interfacial adhesion which led to failure of the laminate. The flexural modulus for dry and seawater aged composite laminates is seen in Figure 16b. In dry condition, the G laminate had higher flexural modulus than B laminate. Furthermore, the flexural modulus of hybrid laminates increased with addition of glass fiber content. This was because the glass fiber reinforcement increased the stiffness of the hybrid composites. Results from tensile modulus of the hybrid composites, however, were not affected by the stacking arrangements and remained fairly consistent. There was a reduction in flexural modulus in G composite compared to B which basically stayed consistent after seawater ageing. The rate of reduction in modulus for GB1, GB2, and GB5 after ageing was fairly consistent. However, GB4 specimen had a substantially lower reduction in modulus than GB2 and GB3. This could be due to the higher water absorption rate of the outer basalt fiber layer.

#### Flexural Failure Analysis

**Figure 17:**
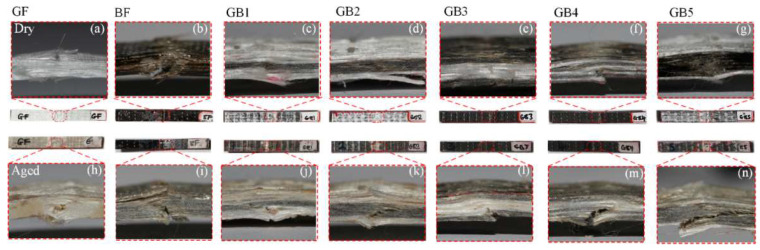
Photograph of flexural test specimen in dry (**a**) GF (**b**) BF (**c**) GB1 (**d**) GB2 (**e**) GB3 (**f**) GB4 (**g**) GB5 and seawater aged condition (**h**) GF (**i**) BF (**j**) GB1 (**k**) GB2 (**l**) GB3 (**m**) GB4 (**n**) GB5.

Figure 17 shows the failure morphologies of dry and seawater aged specimens. The general damage morphology after flexural testing usually resulted in compressive failure from the top, tensile failure from the bottom of laminate and shear or delamination, which took place within the specimens. However, the failure mode in the present experiment showed most of the specimen displaying macro-crack and kink bands in the compressive zone which gradually spread across the width of the specimen. Similar failure phenomena were also reported in [18].

From Figure 17a,b, it can be seen that BF damage is more prominent than GF, due to its slightly lower strength and higher failure strain, as seen in Figure 17a and Table 3. The damage of GB1, GB2 and GB5 seen in Figure 17c,d,g hybrid composites was restricted by the outer glass fiber layers having lower strain and high stress capacity. This enabled these groups of hybrids to attain an increased strength and strain than plain laminates. From Figure 17e,f, GB3 and GB4 hybrids with higher strain fibers on the outer part, had good fracture integrity on the outside in the dry state but lower improvement in strength, as seen in Figure 15a. This outcome showed that for hybrid composites, peak flexural strength was dependent on fiber distribution and sample thickness. A study by Kretsis et al. [32] showed that under flexure, lower strain fibers are bridged by higher strain ones during damage, and this mechanism enables high strength-low strain fibers to achieve their peak strength. Similar conclusions were reached by Zhang et al. [31].

A more severe form of these damage modes was observed in seawater aged composites in general, due to degradation of composite interface caused by hydrolysis and plasticization of the matrix as seen in Figure 17h–n. However, the damage of GB1, GB2 and GB5 hybrid composites shown in Figure 17j,k,n was more in the core area than the outer fiber, while the fractured GB3 and GB4 hybrids seen in Figure 17l,m could be seen from the outer layers. This could be due to the increased modulus of the plain BF after ageing, as seen in Figure 16b.

**Figure 18:**
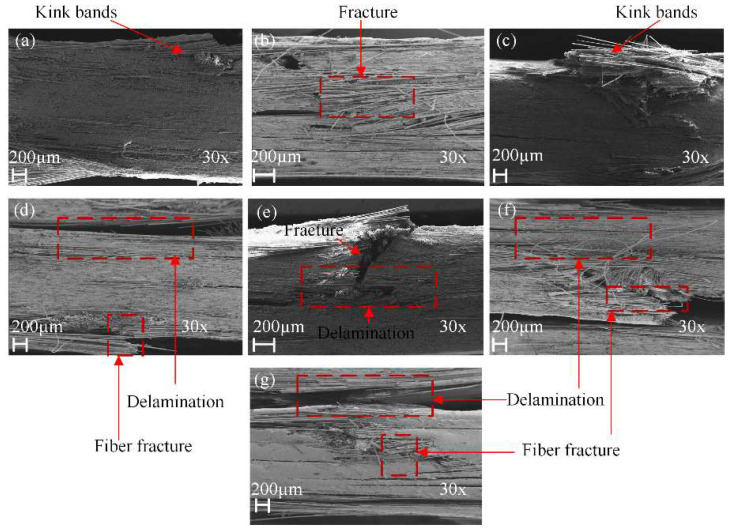
SEM images of dry flexural specimens (**a**) GF (**b**) BF (**c**) GB1 (**d**) GB2 (**e**) GB3 (**f**) GB4 (**g**) GB5.

Figure 18 shows the SEM fracture morphologies of specimens before and after seawater treatment. On the tensile side of plain G specimen, no major crack was formed, as seen in Figure 18a. However, the formation of some kink-bands and micro-buckling were observed with the resistance of laminates to delamination, as well as to compressive damage. In the case of plain B composite, it was fairly evident, as seen in Figure 18b that the presence of fiber fractures and transverse cracks were on the compressive side of the specimen. For the GB1, GB2 and GB5 composites, with external glass fiber layers, delamination and formation of kink-bands were within the compression layer, as seen in Figure 18c,d,g. Meanwhile delamination and fracturing of the basalt fiber core was observed with increase in core thickness, respectively. GB3 and GB4 specimens showed delamination of the core and fracturing of the compressive side, as seen in Figure 18e,f.

**Figure 19:**
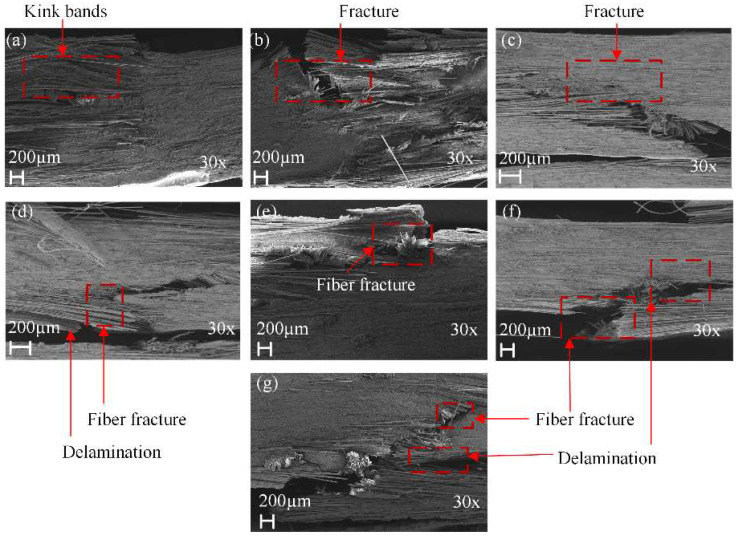
SEM images of seawater conditioned flexural specimens (**a**) GF (**b**) BF (**c**) GB1 (**d**) GB2 (**e**) GB3 (**f**) GB4 (**g**) GB5.

Figure 19 shows the fracture morphologies of seawater aged specimens. In Figure 19a,b, the failure mode of plain G and B specimens after seawater ageing were fiber fracture, formation of kink-bands and micro-buckling of fibers. However, fracturing of fibers were more severe in B composites. The damage morphologies of GB1, GB2 and GB5 hybrid composites after seawater treatment were further fracturing of the core area and separation of fibers due to interface degradation, as seen in Figure 19c,d,g. Severe fracturing of GB3 and GB4 hybrid composites after seawater ageing was mostly observed in the compressive zone, as seen in Figure 19e,f.

## 4. Conclusions

A study concerning the effect of seawater treatment on the mechanical properties of plain and hybrid glass/basalt reinforced composites in various configurations was presented. Specimens were fabricated via a vacuum bagging process, after which their tensile, flexural and charpy impact properties were obtained prior to, and after, ageing. The failure modes of dry and seawater aged specimens were examined through SEM. From the test findings, the main conclusions are as follows:

1. The tensile strength of alternately sequenced hybrid composite (GB4) was the highest in dry condition with 13% strength increase over G composite and 6.63% improvement over B composite. The tensile strength, however, decreased by 13% when aged in seawater, which was an improvement over plain G composite but not B composite. The tensile modulus increased in comparison to both plain composites in dry condition, and, besides, there was an increase among all hybrid composites after ageing. Overall, although GB4 composite possessed the highest tensile strength among the specimens, GB3 retained the highest strength after ageing due its basalt fiber exterior plies. This outcome gives GB3 an edge over GB4 for use in marine application. However, for regular non-marine applications, where strength improvement is paramount, the opposite is true.
2. The flexural strength of GB5 hybrid composite was the highest in dry condition with 48.9% strength increase over G laminate and 76.35% improvement over B specimen. After ageing, the flexural strength decreased by 34%, which represented an improvement over the plain G specimen but not B specimen. The flexural modulus improved compared to plain composite in dry condition; however, there was a decrease among all hybrid composites after ageing. GB5 composite possessed the highest flexural strength. Meanwhile, similar to the tensile test result, GB3 laminate still maintained the highest residual strength in flexure after ageing.
3. The impact strength of dry GB4 composite was the highest at 395.32 kJ/m^2^. Other hybrid architecture, like GB1 and GB5, had higher impact strength than both plain laminates, hence having potential use in certain applications. Unlike the tensile and flexural properties, impact strength retention had an opposite trend. G laminate retained a higher impact strength than B laminate after ageing while GB4 had the highest residual impact strength after seawater ageing.
4. Damage analysis from the different hybrid configurations showed more progressive failure than for the plain laminates. The different fiber sequencing provided a toughening effect by preventing quick propagation of cracks through the composites. In seawater ageing hybrid laminate with basalt exterior plies was more effective in retaining its tensile and flexural properties due to the chemical stability of basalt fibers.

The overall mechanical properties and failure morphologies of some hybrid configurations had superior properties in dry and seawater aged conditions. These improvements open up the possibilities for exploring the afore-mentioned hybrid configurations in the marine environment.

## Figures and Tables

**Table 2:** Material specifications provided by the manufacturer.

Material Name	Surface Mass (g/m^2^)	Density (g/m^3^)	Tensile Strength (MPa)	Elastic Modulus /GPa	Thickness /mm	Elongation (%)
Glass fabric	300	-	1500	72	0.118	2.5
Basalt fabric	300	-	≥2000	≥90	0.115	≥2.0
Vinyl ester	-	1.14	86	3.2	NA	5–6

**Table 3:** Mechanical properties of plain and hybrid specimen before seawater ageing.

Mechanical Properties	Specimens						
	GF	BF	GB1	GB2	GB3	GB4	GB5
Tensile strength/MPa	815.79	864.56	774.47	744.73	810.94	921.91	895.92
Strength retention/%	100	100	100	100	100	100	100
Tensile modulus/GPa	18.3	19.2	17.3	18.2	19.3	20.3	20.3
Flexural strength/MPa	553.08	467.28	730.44	632.04	585.72	567.72	824.04
Strength retention/%	100	100	100	100	100	100	100
Flexural modulus/GPa	24.6	18.1	26.5	24.7	26.7	26.8	26.1
Impact strength/KJ/m^2^	230.45	261.69	317.6	243.86	396.32	222.15	297.23
Strength retention/%	100	100	100	100	100	100	100

**Table 4:** Mechanical properties of plain and hybrid specimen after seawater ageing.

Mechanical Properties	Specimens						
	GF	BF	GB1	GB2	GB3	GB4	GB5
Tensile strength/MPa	646.04	789.93	683.67	589.12	812.10	804.01	650.15
Strength retention/%	79	91	88	79	100	87	73
Tensile modulus/GPa	21.3	19.9	19.2	19.8	20.8	20.6	20.7
Flexural strength/MPa	214.68	390	415.2	525.96	507.24	381.6	539.76
Strength retention/%	39	83	57	83	87	67	66
Flexural modulus/GPa	20.1	19.2	24.9	23.7	16.7	23.8	23.1
Impact strength/KJ/m^2^	212.3	132.11	262.44	216.17	283.63	266.46	210.38
Strength retention/%	92	51	83	89	72	120	71

## Data Availability

Authors are not authorized to share data due to privacy reasons.
